# Typification of *Juniperus pingii* W.C.Cheng (Cupressaceae)

**DOI:** 10.3897/phytokeys.170.59775

**Published:** 2020-12-10

**Authors:** Yong Yang, Jean Hoch

**Affiliations:** 1 College of Biology and Environment, Nanjing Forestry University, 159 Longpan Road, Nanjing 210037, Jiangsu, China Nanjing Forestry University Nanjing China; 2 Domaine de Bonnefontaine, 67260 ALTWILLER, France Domaine de Bonnefontaine Altwiller France

**Keywords:** China, conifer, Cupressaceae, *Juniperus pingii*, typification

## Abstract

W.C.Cheng validly published the name *Juniperus pingii* W.C.Cheng in 1944 by providing a validating Latin diagnosis in de Ferré (1944), but he failed to cite any specimen. He repeated the publication of the name in 1947 with the same Latin diagnosis; he thus published an isonym “*J. pingii*” under Art. 6 Note 2. Cheng (1947) lectotypified the name *J. pingii* when he designated *W.C.Cheng 1015* as the “type” of the isonym. Farjon (2005) overlooked this early designation and his lectotypification of the name with the illustration from the 1944 protologue is not effective as the *W.C.Cheng 1015* specimen is extant.

## Introduction

Cheng (1939) did not validly publish the name “*Juniperus pingii*” because he did not provide a Latin diagnosis for the species. The name was validly published in an article by de Ferré (1944) in which a species diagnosis was provided including a simple figure and a short paragraph of Latin diagnosis in the footnote. Cheng (1947) apparently was not aware of the valid publication of the species name in 1944 and, in 1947, repeated the publication of this species as a “sp. nov.” by providing a Latin diagnosis. We made a comparison between the two publications and found that the diagnosis of 1947 is completely identical to that of 1944. Obviously, the Latin diagnosis in both publications was provided by W.C.Cheng. De Ferré (1944) also ascribed the diagnosis to W.C.Cheng when she wrote “Voici sa diagnose latine telle qu’elle est contenue dans l’ouvrage inédit de W.C.Cheng” (Here is his Latin diagnosis as it is contained in the unpublished work of W.C.Cheng); de Ferré also wrote “*J. Pingii* Cheng sp. nov.”. As a result, the name is to be attributed to Cheng alone and correct citation of the species name is *Juniperus pingii* W.C.Cheng. The two publications are different with respect to type designation: no specimen was cited in the 1944 publication, but Cheng cited two collections in 1947 and designated *W.C.Cheng 1015* [China. Sichuan, Jiulong Xian (“Sikang, south of Chui-lung-hsien”), alt. 2800-3400 m] as the type. Cheng (1947) also cited *W.C.Cheng 939* from the same locality as a paratype. *Juniperus pingii* W.C.Cheng was validly published in 1944. “*Juniperus pingii* W.C.Cheng (1947)” is simply a later isonym (Art. 6 Note 2, Turland et al. 2018) of the original *J. pingii* and Cheng (1947) must be considered to have lectotypified *J. pingii* W.C.Cheng (de Ferré 1944). In *Flora Reipublicae Popularis Sinicae*, W.C.Cheng & W.T.Wang made a new combination [*Sabina pingii* (Cheng) W.C.Cheng & W.T.Wang, as “*Sabina pingii* (Cheng ex Ferré) W.C.Cheng & W.T.Wang”] based on *Juniperus pingii* Cheng [as “*Juniperus pingii* Cheng ex Ferré”] and indicated that the type was collected from Jiulong Xian of Sichuan of China (Wang et al. 1978), suggesting that W.C.Cheng accepted *W.C.Cheng 1015* from Jiulong Xian as the type of the name.

**Figure 1.**
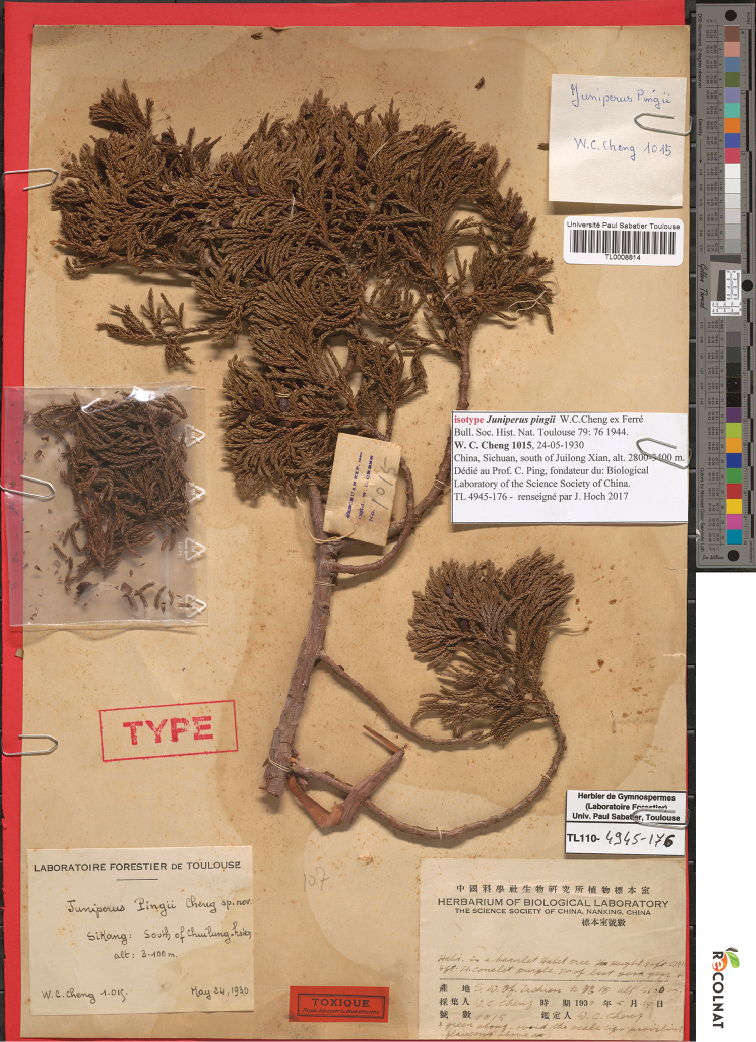
Lectotype of *Juniperus pingii* W.C.Cheng: *W.C.Cheng 1015* (TL0008814).

Both the figure provided in 1944 and the specimens cited in 1947 should be considered as original material studied by W.C.Cheng. Farjon (2005, 2010) overlooked the isonym of Cheng (1947) and, thus, had no idea of the lectotypification of the name. Farjon (2005) lectotypified the name with the illustration in de Ferré (1944), which should be accepted, provided the lecotype designated by W.C.Cheng (*W.C.Cheng 1015*) is lost. However, this is not the case. Cheng (1947) did not indicate the herbarium/institution for the lectotype in his designation. W.C.Cheng had worked in Nanjing until he moved to Beijing in 1962 and most of his specimens were moved to the Herbarium (PE) and were digitised and available online. We did not find *W.C.Cheng 1015* in either CVH (Chinese Virtual Herbarium, http://www.cvh.ac.cn/) or NSII (National Specimen Information Infrastructure, http://www.nsii.org.cn/2017/query.php). W.C.Cheng studied in France and worked with H.M. Gaussen on his Ph.D. thesis “*Les Forets du Se-Tchouan et du Si-Kang Oriental*” (Ma 2011). We finally located the specimen *W.C.Cheng 1015* (TL0008814, Fig. 1) in Université Paul Sabatier Toulouse. This specimen is marked with “Type” and it was clearly studied by W.C.Cheng because it has a label with Cheng’s handwriting “*Juniperus pingii* Cheng sp. nov.”.

We checked the protologue in de Ferré (1944) and found that the illustration (“figure 21” in de Ferré 1944) contains a female cone and a separate seed, both from the lateral view. This figure is too simple to assist identification; it does not show any of the diagnostic characters as Cheng indicated “this species is closely related to *J. recurva* Buch.-Hamilt., from which it differs chiefly in the shorter leaves with distinctly keeled lower surface”. Nevertheless, the actual specimen *W.C.Cheng 1015* possesses diagnostic characters assisting the identification. As a result, there is no reason to supersede Cheng’s lectotypification (*W.C.Cheng 1015*) with Farjon’s designation (the illustration “figure 21” in de Ferré 1944).

## Typification

### Juniperus pingii

Taxon classificationPlantaePinalesCupressaceae

W.C.Cheng in Y. de Ferré, Bull. Soc. Hist. Nat. Toulouse 79: 76, f. 21 (1944)

7991C526-EA51-5A56-AB53-57F37D2C7850

≡ Juniperus pingii W.C.Cheng, Trav. Lab. Forest. Toulouse V, 1 (2): 93 (1939), *nom. inval.*; Juniperus pingii W.C.Cheng, Res. Notes Forest. Inst. Natl. Centr. Univ. Nanking, Dendrol. Ser. 1: 2 (1947), *isonym*; Sabina pingii (W.C.Cheng) W.C.Cheng & W.T.Wang, Fl. Reip. Pop. Sin. 7: 355 (1978).

#### Type.

China. Sichuan, Jiulong Xian (“Sikang, south of Chui-lung-hsien”), alt. 2800–3400 m, 24 May 1930, *W.C.Cheng 1015* (lectotype: TL0008814!).

## Supplementary Material

XML Treatment for Juniperus pingii
